# The Empoisoned Darts of Venus

**Published:** 1989-02

**Authors:** John R. Guy

**Affiliations:** Medical Archivist, Yeovil District Hospital


### The Empoisoned Darts of Venus: John Warren, MD., A Pioneer West Country Venereologist

**John R. Guy**
Medical Archivist, Yeovil District Hospital

0In 1775 Joseph Priestley issued the first volumes of his *Experiments & Observations on different kinds of Air* [1], the fruit of work which had been occupying him for the previous four or five years [2]. In the appendix to volume II of Priestley's work appeared a letter dated 3rd October 1775 in which the writer asserts that there is "no room to doubt of the great advantages the medical world might derive from [the application of *fixed air*] in putrid diseases..." [3] He details the value in cases of "ulcerated sore-throats" of drinks impregnated with *fixed air*, and of milk treated in this way as a suitable vehicle for the administration of Peruvian bark to children. "It is commonly known" he says, "that physic of every kind is to them peculiarly obnoxious" [4] and he reveals a happy insight into child psychology by his perception of the attraction of a fizzy milk-drink for them.

The letter is signed "John Warren, M.D., of Taunton". Then as now Taunton was the county town of Somerset in the south-west of England, a market town at the heart of a rural area, but with a growing industrial base in this last quarter of the 18th century. Who was John Warren, who claims in his letter to have been "strongly preposessed for many years past in favour of the utility of fixed air in certain medical cases" and that he had written his account in compliance with a request from Priestley himself [5]? In fact he was 29 years of age in 1775, and had taken his M.D. at Edinburgh only five years previously. Nonetheless he could claim, despite his youth and comparative inexperience, to have made his mark on the period of history which Lester King has called "the adolescence of present day medicine" [6].

John Warren had been born in 1746, and had entered the university of Edinburgh at about the time the *Statuta Solemnia* of 1767 were enacted, which systematised the course of study for medical students [7]. Here he was to come under the lasting influence of William Cullen and Joseph black, both without doubt, among the leading physicans in the country, and professors of the Institutes of Medicine and Chemistry respectively. Cullen, whom Warren was to call "professori celeberrimo, praeceptori suo..." [8] kindled his interest in materia medica & fevers; Black a parallel attraction to chemistry. The professor of the Institutes of Medicine departed from tradition and lectured in the vernacular. He took a keen personal interest in the progress of his students, and his influence can be detected in Warren's M.D. thesis, which he successfully defended in July 1770, "de Cortice Peruviano...", a thesis which was published in the same year [9]. In 50 pages, Warren examines the effects and effectiveness of 'Peruvian bark' in fevers, phthisis, and other complaints. He acknowledges the work of Thomas Sydenham, John Pringle, George Cleghorn, Thomas Percival and John Gregory, among many others—including, of course, Cullen. Pringle & Cleghorn's emphasis upon careful clinical observation, their "orientation to current writers rather than to the wisdom of the ancients" [10], Gregory & Percival's insistence upon the humane and sympathetic treatment of patients, all seem to have struck a responsive chord in John Warren.

Joseph Black, by contrast, was responsible for Warren's interest in "fixed air". Black's experiments had led to his discovery of the gas he named "fixed air"—familiar to us as carbon dioxide [11] —and Warren, of whom an anonymous contemporary wrote his "chemical abilities are everywhere conspicuous" seems to have continued the research. As can be seen from his letter to Priestley, his interests in fixed air, in Peruvian bark and in fevers became blended together in his clinical practice. It is, incidentally, just possible that Priestley & Warren were personally acquainted. Both were Unitarians, and could have met at the earl of Shelburne's seat at Calne, during Priestley's residence there in the early 1770s.

On graduation from Edinburgh, Warren seems to have gone to France, where he continued his work on fevers, and completed investigations, begun in Scotland, into the prevention and cure of venereal disease. He was to publish—in French—*A New Method of curing and preventing the virulent gonorrhea*... which appeared in an English translation in 1771 [13]. This is a work of considerable interest. Warren, like Francis Balfour and other Scottish clinicians—though not John Hunter, after his unfortunate experiment—was convinced of the duality of gonorrhea and syphilis [14]. The new 'remedy' for the former, which he discusses in his 1771 work, involved the injection into the male urethra of a solution of an alkaline salt in a caustic state, with added camphor [15]. The injection of a prepared mixture in this way had been advocated in cases of the *chaude pisse* as far back as the 14th century by John Aderne [16]. Warren after a period of experimentation [17] had come to the conclusion that "the venereal virus is a matter lodged in the urethra [which] acts as a ferment assimulating the natural mucus, and by its acrimony irritates & inflames every part with which it comes into contact" [18]. His caustic alkali solution could dissolve mucus, & its injection into the urethra he believed "brought out" the virus, as well as giving the urethra "the tone necessary to resist the disease" [20]. Warren supported his claim by instancing sixteen "young men of my acquaintance" who, having contracted gonorrhea, he had treated in this way —14 had been cured [21]. He also mentions a colleague in Scotland who seems to have anticipated Hunter, for he had put "venereal virus from an infected person" into his urethra, & after four days had developed symptoms. Warren's treatment had cured him in 24 hours [22]. The syringe, he says, has to be induced half an inch into the urethra, & the solution injected gently. The application was to be repeated four times, within two minutes of each other, and occasionally repeated after 12 hours [23]. But he sounds two cautionary notes. The treatment had to be given within two days of infection appearing "else it increased the disease" [24] and it was no good for women—"the parts to which it is applied have too large a surface". [25] Warren concluded, in a somewhat purple passage, by advocating the prophylactic use of his treatment. Those who were incapable "of resisting the empoisoned darts of Venus" should always carry with them "those defensive arms that are so necessary for such as depart from the paths of virtue" [26]. A kit comprising "a small phial of the caustic alkali dissolved, a box of pomatum & a syringe" should be in the luggage of every such gentleman, and application made immediately after sexual intercourse [27]. Urethral injection for gonorrhea does seem to have become an accepted treatment for a time [28].

By 1775 Warren had established himself at Taunton, as one of the two physicians in practice there. Both were Edinburgh graduates [29]. Here he settled down, married, produced a family, and became a prominent member of the Mary Street Unitarian Church. Among his patients—and those he accounted friends—were the Countess of Chatham, and her son, William Pitt the younger, England's Prime Minister. Their country estate was close to Taunton [30].

Then, about 1788, Warren's health broke down. Although he nowhere says so, pthisis is the most likely diagnosis. In the autumn of 1788, in an attempt to recover his health, he set out for Lisbon. His surviving letters to a Taunton surgeon [31] give some insight into the life of an English invalid in the city, and his impressions of it. He nearly failed to arrive. The journey from Taunton to Falmouth drained his strength [32], and during the 12 day voyage he and his wife experienced becalming and a terrible storm [33]. Among his travelling companions were the Portuguese Ambassador to the Court of Vienna, & the son of the Prime Minister, possibly the marquis of Angeja, who died in 1788, or José Seabra da Silva, who took office under Maria I about the time Warren arrived in Lisbon [34]. Both were "very courteous and obliging" and the former Warren described as "the best bred man I ever met with" [35]. Other fellow travellers were not so fortunate. One, a girl of 19, "a most beautiful creature" travelling with her father & suffering from pthesis, lived only long enough to be taken on shore "and to wonder that the air of Lisbon did not afford her immediate relief" [36].

Warren established himself at "Williams's or the English Hotel" overlooking the Tagus, & delighted in the view from his windows [37]. Every day he rode out on a mule, a favoured resort being "the celebrated aqueduct of Alcantara, which is a most stupendous monument of modern architecture" as he reported [38]. Like most Englishmen he grumbled about the weather [39], but spent much time resting and talking with what seems to have been a group of regular callers. He was even well enough at first to help Dr Hair, "the first & only physician of any practice among the English in Lisbon" with some of his patients [40]. Lisbon itself did not impress him, nor did some of its inhabitants. He claimed, in a letter of 20th February 1789 that "at every door [are] men, women and children lolling on the ground, placing their heads by turns in each others' laps to have them 'abridged of the Retinue' that may be contained there". He also wrote of monkeys trained to carry out the same activity, and "remarkably dextrous in their professional pursuits". They were hired out for what was quite literally a capitation fee [41].

By this time Warren was evidently depressed. He was weaker, describing himself as "in an enfeebled and emaciated state" and by now having to be driven in a carriage for his "airings" [42]. He was not to recover, and, according to his memorial [43] died on 12th November 1789, nine months after this last surviving letter home from Lisbon. He was 43. He is forgotten now, but for a few brief years in the 18th century he epitomised the educated and enlightened English provincial physican. His life in Edinburgh, Taunton and Lisbon also, perhaps, is an example of the 'British-Portuguese Connection".

### REFERENCES

1. Joseph Priestley, *Experiments & Observations on different kinds of Air*. (3 vols, London, 1775)
2. Jack Lindsay (introd.), *Autobiography of Joseph Priestley*. (Bath, 1970) p 20.
3. *Experiments & Observations*..., II, p 375.
4. ibid., p 376.
5. ibid., p 375.
6. Lester S King, *The Medical World of the Eighteenth Century*. (Huntington, N.Y., repr. 1971), p xvi.
7. Sir Alexander Grant, *The Story of the University of Edinburgh during its first Three Hundred Years*. (vol. 1, London, 1884) pp 330–332.
8. The dedication of Warren's M.D. thesis of 1770.
9. *Dissertatio Medica Inauguralis de Cortice Peruviano... pro gradu doctoris... Joannes Warren, Britannus, 12 July 1770*. (Edinburgh, 1770).
10. King, op.cit., p 137.
11. Douglas Guthrie, *A History of Medicine* (London, 1945) p 261. cf. W Ramsay, *Life of Joseph Black* (Glasgow, 1904).
12. The translator, identified only as 'A Surgeon', of Warren's work on gonorrhea (q.v.), p vi.
13. *A New Method of curing and preventing the virulent gonorrhea, to which is added a chemical investigation of a remedy called, The Preservative Antivenereal Water, written originally in French by J Warren, M.D. of the University of Edinburgh*. (London, 1771). Warren was later a Corresponding Member of the Royal Medical Society of Paris.
14. W A Pusey, *The History & Epidemiology of Syphilis* (Springfield, III., 1933) p 44.
15. *New Method*... pp 10 & 11.
16. D'Arcy Power, 'Venereal Diseases', in W R Bett, *A Short History of Some Common Diseases* (Oxford, 1934), p 36.
17. detailed in *New Method*...,pp 9–15.
18. ibid., pp 18,19.
20. ibid., pp 19 & 21.
21. ibid., p 24. He also mentions 20 other cases, all successful, on p 25.
22. ibid., p 24
23. ibid., pp 35 and 36.
24. ibid., pp 24–25.
25. ibid., p 34.
26. ibid., p 38.
27. ibid.
28. cf. King, op.cit., p 313 for the treatment of gonorrhea in a woman by injection [which Warren had said would not be effective] in 1787 or 1788. It is not clear whether this injection was urethral or merely vaginal.
29. John R Guy. *Malachi's Monument. The Taunton & Somerset Hospital at East Reach*. (Crewkerne, 1987) p 15. The other physician was John Cabell, who had graduated M.D. from Edinburgh in 1755.
30. In one of his letters Warren speaks of the "proofs of friendship & attention" shown him by Lady Chatham, & "the great condescension & kindness of Mr Pitt". (Letter in the Somerset Record Office, dated from Falmouth, 14th October 1788).
31. Woodforde. A relative of the clerical diarist, the Revd. James Woodforde.
32. Letter dated 14th October 1788.
33. Letter dated November 1788. Their vessel was "left to the fury and to the mercy of the elements, and during this period... was driven before the wind, in a retrograde course, at the rate of 15 knots per hour".
34. H V Livermore, *A New History of Portugal* (Cambridge, 1969) pp 239, 243.
35. Letter dated November 1788.
36. ibid. She was one of 10 out of 11 children that the father, a Mr Farquarson, had lost, "almost all of them in the same direful disease".
37. ibid. The Tagus he said, was "the finest river in the world" and "the tout ensemble [formed] the most enchanting prospect I ever beheld".
38. ibid.
39. "oppresively warm" in November, "70 degrees, which is summer heat in England".
40. Letter dated 20th February 1789. He was careful to avoid cases which might involve him in calls "at unreasonable hours or in unfavourable weather" & perhaps largely confined his attentions to other "invalids" at Williams's—one of whom gave him £20 for his services.
41. ibid.
42. ibid.
43. In the Mary Street Unitarian Church in Taunton, Somerset.

